# Advancing undergraduate medical education regarding the care of transgender and gender Diverse persons and communities

**DOI:** 10.1007/s40037-022-00732-w

**Published:** 2022-11-26

**Authors:** Carl G. Streed Jr, May Navarra, Jorden Klein

**Affiliations:** 1 Center for Transgender Medicine & Surgery, Boston Medical Center, 02118 Boston, MA USA; 2 Section of General Internal Medicine, Department of Medicine, Boston University Chobanian & Avedisian School of Medicine, 02118 Boston, MA USA; 3 Division of Emergency Medicine, Department of Medicine, University of Toronto, M5S 1A1 Toronto, Ontario Canada

Currently, there is no clear consensus regarding the format or integration of educational interventions to improve learner competencies in transgender and gender diverse (TGD) health. Comprehensive medical education resources, based on Association of American Medical Colleges (AAMC) competencies [1], have been developed and piloted in various contexts [2, 3]. What is needed is a clear framework of core values, objectives, and principles, as well as a guide flexible enough to be implemented across various institutions with differing timelines and formats for their particular curriculum.

The increasingly urgent need for such frameworks regarding undergraduate medical education (UME) training on TGD health cannot be overstated. A growing proportion of youth and adult populations identify as TGD [4, 5]. For example, approximately 0.33% of Canadians aged 15 years and older identify as TGD [6]. These populations face a multitude of interpersonal and societal stressors that contribute to inequities in healthcare access, and morbidity and mortality [7–9]. While the number of community health centers focused on sexual and gender minority (SGM) health have been adding more programs to serve TGD persons, these services are geographically dispersed and limited in what services are available to meet the unique needs of TGD persons [10]. Further, TGD healthcare is siloed into specialist programs (e.g., endocrinology, plastic surgery, etc.) to the point that other clinicians view TGD health as specialist care, absolving themselves of the obligation to develop basic competencies in this area. And while sociopolitical advances have improved some legal protections and rights, TGD persons and communities continue to face a discriminatory healthcare system that inconsistently provides competent, comprehensive care [11].

While research has demonstrated a clear association between transphobia and the acquisition of clinical knowledge regarding the care of TGD persons [12], the dearth of TGD-focused medical education content must be addressed, whether by school leadership, accrediting bodies, or national and international organizations (e.g., World Health Organization) [13]. A 2009–2010 survey of allopathic and osteopathic medical schools in the US and Canada revealed that less than a third addressed gender-affirming medical and surgical interventions [13]. Subsequent evaluations of graduate medical education (GME) have revealed little change in UME on the topic. A 2016–2018 assessment of an online module on SGM health, including material on TGD health issues, found no variation in pre-test knowledge among 1018 residents regardless of postgraduate year, suggesting no variation in UME training over the three-year study period [14].

Approaches taken by UME and GME leadership to providing learners with the knowledge and skills to care for TGD persons vary significantly across institutions, reflecting competing priorities, resources, and educator preparedness and competency in addressing TGD health issues [2, 15]. Furthermore, while The Joint Commission [16] and US Department of Health and Human Services [17] released comprehensive plans to improve SGM health, the Liaison Committee on Medical Education, AAMC, and Accreditation Council on Graduate Medical Education do not require the inclusion of gender-affirming care nor broader TGD health content for accreditation, medical student instruction, or graduate medical training, respectively [18]. Consequently, the determination of how best to implement curricula regarding TGD health, let alone the decision on whether to implement such a curriculum at all, is left to individual UME institutions, who face a number of barriers to integrating such curricular content: lack of training opportunities, absent faculty expertise and training, and a packed curriculum [15].

To facilitate the inclusion of TGD health content into UME training, Ellaway et al. published “*An undergraduate medical curriculum framework for providing care to transgender and gender diverse patients: A modified Delphi study*” in 2021, which brought together learners, educators, and TGD community members to develop a framework for teaching the healthcare needs of TGD persons and communities [19]. After recognizing the paucity of guidance on teaching TGD health issues in UME, the authors generated a framework through a modified Delphi process. The modified Delphi process included four rounds: 1) terminology and syllabus, 2) clarifying results from Round 1 and exploring how TGD content should be taught, 3) clarifying results from Round 2 and addressing safety, attitudes, and intersectionality regarding TGD care, and 4) providing feedback on provisional recommendations and areas with low consensus. Following four Delphi rounds and the completion of a draft syllabus and curriculum through the framework, TGD community input was sought. TGD people rightly highlighted the need to ensure that TGD health is mandatory and not content that learners can opt out of due to “conscientious objection”; to do so validates the moralization of trans identities and TGD people and perpetuates the current system of tiered training and subsequent care of TGD people.

Notably, TGD community members were not included in the Delphi phase of the study to focus on the UME teaching material rather than delivery of care for medical services. Although this approach may be practical for curriculum building, TGD feedback should be included at all stages of development. Possible alternatives to include TGD voices earlier are to sample TGD persons in medicine for the Delphi rounds or to include the TGD community voices earlier and document healthcare-related grievances and successes for future interventions. Additionally, the iterative Delphi process employed by Ellaway et al. allowed for multiple opportunities to narrow and explore the key concepts critical to caring for TGD persons and providing gender-affirming care. Their approach, while comprehensive, highlights opportunities for improvement in the development and implementation of UME curricular change that addresses TGD health. Early inclusion of TGD persons, particularly TGD healthcare providers and TGD experts in community care, would have strengthened the Delphi process [18]. For too long, TGD care has been governed by cisgender gatekeepers; medical education reflects this with a preponderance of content that has pathologized TGD identities. However, Ellaway’s final framework—with TGD community input—does provide clear guidance on core values, teaching objectives, and teaching principles to advance UME regarding TGD health.

What remains to be presented by Ellaway et al. is a guide for when, during their UME education, learners should receive specific knowledge and skills germane to the care of TGD people and communities. Such a guide would aid UME leaders in more rapidly incorporating this guidance. However, it is worth noting that the development of such a guide is complicated by the current debate in UME and, to a lesser extent, GME around using a competency-based model of advancement that could accelerate learners’ progression through any phase of their training. Under a competency-based model, how would the framework by Ellaway et al. be utilized to ensure adequate training occurs in a potentially abbreviated UME timeline? While a competency-based model has the potential to graduate larger numbers of clinicians, there is potential with an already packed set of curriculum requirements that additional recommendations specific to TGD health and well-being could be cut.

Through years of various curricular advancements and re-designs, the need to prepare learners to deliver high-quality, person-centered care remains consistent, and Ellaway’s framework offers a hopeful look forward into the future of medical education. Developing working competencies in caring for TGD patients involves more than didactic knowledge on gender-affirming medical or surgical care; it also requires the acknowledgement of how transphobia in medicine perpetuates real, immediate harms that precipitate morbidity and mortality, and prepares learners to champion the humanity of TGD people in solidarity. We look forward to the incorporation of more, or any, TGD health content in UME as well as in graduate and continuing medical education.
